# Long-Term Viscoelastic Behavior of Polyisobutylene Sealants before and after Thermal Stabilization

**DOI:** 10.3390/polym16010022

**Published:** 2023-12-20

**Authors:** Urška Gradišar Centa, Alen Oseli, Mohor Mihelčič, Aleš Kralj, Matjaž Žnidaršič, Miroslav Halilovič, Lidija Slemenik Perše

**Affiliations:** 1 Faculty of Mechanical Engineering, University of Ljubljana, Aškerčeva 6, 1000 Ljubljana, Slovenia; 2 Reflex Gornja Radgona d.o.o., Podgrad 4, 9250 Gornja Radgona, Slovenia

**Keywords:** frequency–temperature superposition, polyisobutylene, relaxation time spectrum, structural changes, van Gurp–Palmen plot, viscoelastic properties

## Abstract

Polyisobutylene (PIB) is commonly used as a primary sealant in multi-layer insulating glazing elements, where temperatures often exceed 100 °C. At such conditions, PIB undergoes structural changes, causing different relaxation dynamics and leading to decreased lifetime of the material. Understanding thermal behavior is therefore imperative for achieving effective insulation of these materials for long-term use in insulating application. The present study was focused on the temperature dependence of viscoelastic behavior of two commercially available polyisobutylene (PIB) materials, which are commonly used as primary sealants for energy-efficient multi-layer glazing units. The long-term viscoelastic behavior of the materials before and after thermal treatment at high temperatures was studied by using time–temperature superposition (tTS). Van-Gurp–Palmen plots were obtained directly from experimental data and enabled the study of thermally induced changes, while the relaxation time spectra were calculated from master curves and enabled the calculation of molecular weight distribution. The results showed that, after thermal treatment, the structure of PIB materials changes from linear to branched, while the molecular weight distributions transition from monomodal to bimodal. The untreated samples exhibited viscous-like behavior, while the thermally stabilized samples exhibited solid-like behavior, extending the material response for ~6 decades towards a longer timescale. Moreover, the presented results can be directly used to simulate the mechanical responses of the sealants using currently available FEM software packages to predict their functional and structural lifetime.

## 1. Introduction

In accordance with global sustainable development [1], European green deal [2], and ecological guidelines, the most important social objective is to minimize energy consumption and consequently lower the carbon footprint. These goals are very important when planning the construction of new and renovated residential buildings. The new “green” urban style of building construction, the so-called “zero-emission buildings”, include an ever-increasing proportion of the outer insulating window elements that ensure natural luminance (sunlight) in sufficient intensities [3]. Lam et al. [4] reported that in tropical regions the lighting instruments represent as much as 20–35% of total electrical energy expenditure in buildings. In other regions, this proportional quota is even higher. However, for the high energy efficiency of modern buildings, the reduction in heat losses is crucial, or, in another words, the thermal insulation should be improved. One of the major contributions of energy efficiency is using high-performance multi-layer windows [5], where choosing the optimal properties of each component, such as the glazing gap thickness, gas, frame materials, sealants, and low-emissivity coatings, can significantly improve energy efficiency [6]. It has already been reported in the literature that using six-plane insulating glazing units leads to a 50% reduction in annual energy consumption [7]. The reduction was attributed to the low thermal transmittance of the insulating glazing units, expressed as Ug values, which were calculated to be 0.26 W/m^2^K according to the standard EN 673:2011 [7]. Although a higher number of glass panes improves the overall thermal insulation performance, it complicates its thermomechanical behavior since thermal load becomes more dominant as the number of glass panes increases (the glass panes in the units absorb light, which is converted into heat) [8]. Consequently, the temperature in the intermediate chamber significantly increases, resulting in decreased durability of various window materials. Ensuring longevity in multi-layer insulating structures has been exposed as the key problem since not only the temperature but also pressure and humidity fluctuations can induce structural changes in window materials and consequently lead to loss of desired properties [9,10]. A group of extremely important window materials are sealants, which are highly affected by the temperature, UV light, and other factors, which induce the formation of internal stresses and deformations, such as the displacement of thermoplastic sealants resulting in various defects, such as twisting, bulging, cracking, out-of-straightness, and out-of-squareness of the edge seal [11]. The resulting changes in cavity pressure exert lateral load on the glass panes and deflect them, which induces deformations in sealants and leads to a reduction in the lifetime and energy performance (or energy estimation of buildings) [12]. Moreover, the above-mentioned factors can lead also to chemical structural changes, affecting the fragility of PIB, which decreases with increasing molecular weight [11]. Due to thermal fluctuations during the sunny and dark parts of the day on a short timescale or summer and winter parts of the year on a long timescale, sealants are also subjected to cyclic mechanical loading due to temperature variations, which can cause differences in mechanical behavior. In the literature, it has been reported that, in the intermediate chamber in two- or three-pane glazing units, the temperature limit at 80 °C is hardly exceeded; however, in the six-pane glazing, it was observed that the temperatures may reach up to 110 °C [13]. With increasing temperature, the vapor permeability in the sealant increases too [7]. This fact indicates high temperature stresses of primary sealant, such as polyisobutylene (PIB). PIB is a synthetic elastomer with thermoplastic mechanical behavior, most commonly used as a primary sealant in the multi-layer insulating glazing elements. PIB has low water and gas permeability, high chemical resistance [14], and extremely low glass transition temperature, i.e., ~−68 °C [15]. However, the mechanical properties of PIB strongly depend on the temperature; i.e., they rapidly decrease with increasing temperature [16]. Therefore, as the temperature in the intermediate chamber of multi-layer glazing units significantly increases and exceeds 100 °C, the PIB undergoes structural changes, causing different relaxation dynamics. There are some studies reported in the literature regarding the effect of sealing system quality on the durability of multi-layer glazing units [17,18]. Notable geometrical changes in the PIB seal in glazing units after cyclic loading have been reported [11] to reduce its service time; however, according to the authors’ knowledge, there are no studies reporting and explaining structural changes in the sealant due to temperature changes. Moreover, there have been some studies reported regarding thermo-oxidation of PIBs by using accelerated heat aging in the 40–100 °C range [19]. In order to increase the lifetime of sealants, the addition of various additives [20] or the use of dual seal systems, like a PIB/silicone system [11], or double sealants, like PIN sealant adjacent to silicone sealant [9], is also reported in the literature. However, according to the authors’ knowledge, there are no recent literature studies available on thorough research regarding the prediction of functional and structural long-term behavior of PIBs by time–temperature superposition with the ability to use the experimental data directly in the currently available FEM software packages.

The aim of the present research was therefore to address the thermal stability of two commercially available PIB materials, which are applied to six-pane multi-glazed units. The study was performed by observing polymer relaxation dynamics, i.e., viscoelastic properties, through a wide temperature range. Time–temperature superposition enabled the prediction of the long-term behavior of the studied materials, while spectral and molecular weight distribution analysis provided insight into the structural (molecular) changes during stabilization.

## 2. Materials and Methods

Two commercially available PIB materials commonly applied in multi-glazing units, marked as PIB 1 and PIB 2, were used for comparable study. The chemical composition and molecular weight of the materials were not disclosed by the producer. The investigation was conducted on untempered (unconditioned) samples marked as (UNT) and tempered (thermally conditioned) samples marked as (T), where conditioning parameters (temperature and time) were selected based on stable viscoelastic response during temperature sweep tests (explained in continuation).

**Figure 1:**
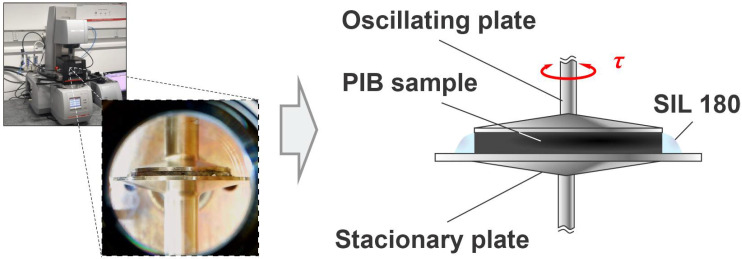
Schematic representation of viscoelastic analysis on PIB materials using plate–plate configuration within modular rheological system MCR 302.

Viscoelastic properties, i.e., storage G′ and loss G″ modulus, were determined through series of rheological measurements in an inert nitrogen atmosphere by using modular rotational rheometer MCR 302 (Anton Paar, Graz, Austria). A parallel plate sensor system with diameter of 25 mm was used for the measurements, where the upper (PP25/S) and the lower Peltier (P-PTD 200) plates were sandblasted to avoid wall slip. The gap between the plates was set to 1 mm and varied with temperature to compensate normal force at 0 N. For all the measurements, the outer edge of the sample in the sensor system was surrounded by silicone oil (SIL 180, ThermoFisher Scientific, Waltham, MA, USA), which prevented drying of the sample and evaporation of individual components from the sample during the measurements (Figure 1).

The temperature sweep tests were performed in the temperature range from 15 °C to 180 °C at constant frequency of 1 Hz and shear stress of 200 Pa for PIB 1 and 250 Pa for PIB 2, respectively, within linear viscoelastic range (LVR). The values of shear stresses in LVR were determined beforehand by amplitude tests, where the amplitude (shear stress) was increasing from 10 Pa to 3000 Pa at constant frequency of oscillation 1 Hz [21].

The frequency sweep measurements were performed at different constant temperatures in the frequency range from 100 to 0.01 Hz, at constant shear stress within LVR, i.e., 1000 Pa for UNT and 200 Pa for (T) samples, respectively. For the UNT samples, the tests were performed at temperatures from −20 °C to 60 °C with increment of 10 °C, while the temperatures for tempered T samples ranged from −20 °C to 180 °C, with the increment of 20 °C. Different temperature ranges were selected for T and UNT samples based on temperature sweep results as they indicated no structural changes under ~80 °C, implying materials’ thermo-rheological simplicity. Thermo-rheological simplicity is important for predicting long-term behavior as it enables the use of time(frequency)–temperature superposition and spectral analysis [22]. However, as the temperature exceeded ~80 °C, structural changes were observed, which broke thermo-rheological simplicity of the material, disabling the use of above-mentioned tools. To determine at which temperature the structural changes stabilize and whether the material regains thermo-rheological simplicity, the temperature range was increased to 180 °C.

Based on the obtained isothermal segments of viscoelastic properties for PIB 1 and 2, master curves (material functions) were constructed at reference temperature of 20 °C by applying frequency–temperature superposition principle with so-called Closed Form Shifting algorithm [23,24,25], which determines the material behavior over a wide frequency (time) range. Furthermore, spectral analysis (material modelling) was performed from generated master curves using Edge preserving algorithm [24] to determine a relaxation time spectrum H(λ). In addition to modelling, H(λ) provides the information of relaxation of molecular chains and serves as an input data for determination of molecular weight distribution (MWD) using various kernels, in the present work, Doi–Edwards kernel was used [26]. Spectral and MWD analysis was performed by using commercially available software for molecular dynamics (RheoCompass, Anton Paar, Graz, Austria).

## 3. Results and Discussion

### 3.1. Temperature Sweep Tests

**Figure 2:**
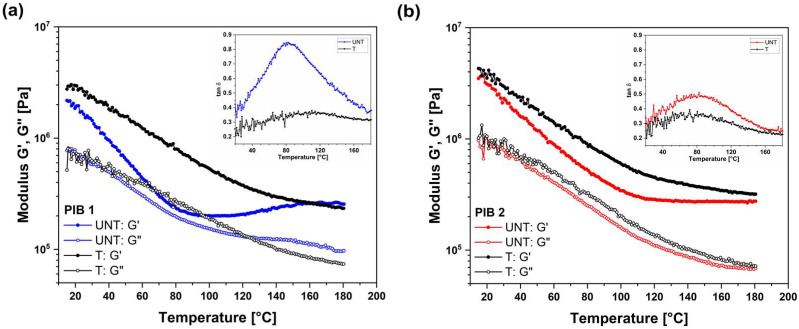
Temperature sweep tests for (**a**) PIB 1 and (**b**) PIB 2 before (UNT) and after (T) structural changes. The inserts present the corresponding damping ratio—tan δ curves.

The comparison of dynamic moduli, i.e., storage (G′) and loss (G″) modulus, for PIB materials before (UNT) and after (T) structural changes is presented in Figure 2. The temperature dependency of dynamic moduli during the first heating from 20 °C to 180 °C is presented for PIB 1 with blue and for PIB 2 with red curves, respectively. For both PIB materials used, the storage modulus (elastic response) decreased with increasing temperature in the lower temperature range studied. The lowest values of G′ were obtained around temperature 90 °C for (UNT) PIB 1 and 100 °C for (UNT) PIB 2, respectively. At these temperatures, structural changes in the materials were induced and the elastic modulus of PIB 1 started to increase, while the elastic modulus of PIB 2 levelled off. On the other hand, the viscous contribution to viscoelastic behavior, (G″), decreased over the entire temperature range for both PIBs examined. However, a more pronounced decreasing was observed in the lower temperature range. After the first heating, the samples were cooled to the initial temperature of 20 °C and heated again in the same temperature range. The second heating is shown in Figure 2 for both samples, presented with black curves. The results show that the structure of the samples in the second heating was changed as the storage moduli of both samples gradually decreased with increasing temperature over the whole temperature range studied, with no minimum detected. Moreover, as the temperature exceeded 140 °C, the values of storage modulus before (UNT) and after (T) structural changes reached values that were almost the same as in the first heating, indicating the stabilization of the structure.

The damping ratio tan δ, defined as the ratio of loss and storage modulus, indicates elastic and viscous contributions to viscoelastic response, here used to identify the critical temperature at which the material undergoes structural changes. By increasing the temperature, the viscous part becomes more important; hence, tan δ increases; however, due to structural changes at the critical temperature, the elastic part increases; thus, tan δ decreases through the remaining temperature range. The critical temperatures were, for both PIB materials, determined as the temperatures where the maximum tan δ curve occurred (inserts in Figure 2). For the (UNT) PIB 1 sample, the maximum value of tan δ was determined at about 83 °C, while, for the (UNT) PIB 2 sample, the maximum tan δ was at about 85 °C. The peaks of tan δ for the tempered (T) PIB 1 and PIB 2 samples were not distinct. Moreover, in the whole temperature range studied, the (T) PIB 1 and 2 samples exhibited lower values of tan δ compared to the (UNT) samples. This indicates that the structure of the tempered samples exhibited a lower viscous contribution to the viscoelastic response, resulting in a more stable structure of these materials.

From the above-mentioned results, we can conclude that the (UNT) and (T) PIB samples exhibited a solid-like viscoelastic behavior, where the thermal stability was improved in the tempered samples. Moreover, since the temperatures in multi-layer glazing elements exceed 80 °C, the material undergoes structural changes. It is reasonable to believe that this also improves the long-term viscoelastic behavior of the sealant. The structure of the PIB material in the insulation glazing units should be stable in order to avoid the unwanted deformations of the sealant; therefore, the isothermal characterization of PIBs from −20 °C to 60 °C (below the temperature of structural changes) and from −20 °C to 180 °C (above the temperature of structural changes) was performed to provide detailed information on the temperature-induced structural changes and long-term viscoelastic behavior of the studied materials.

### 3.2. Frequency Tests of PIBs at Various Temperatures

**Figure 3:**
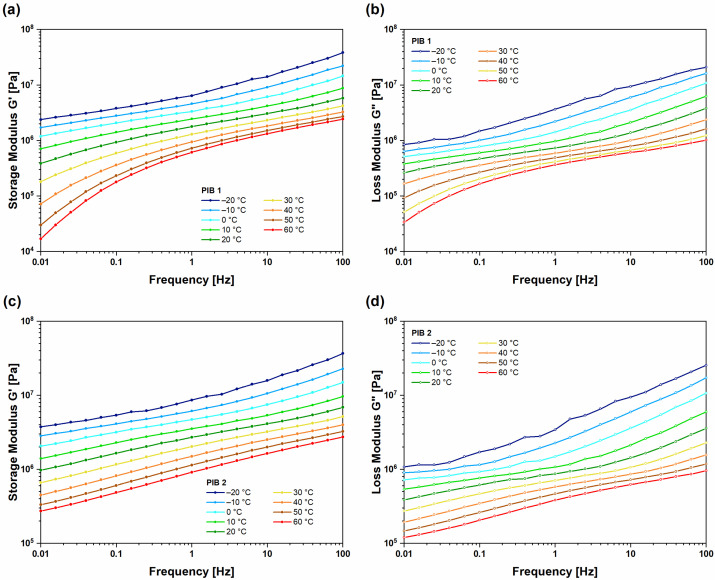
Isothermal segments of frequency-dependent dynamic moduli for (UNT) PIB 1 sample ((**a**) storage and (**b**) loss modulus) and (UNT) PIB 2 sample ((**c**) storage and (**d**) loss modulus).

Figure 3 shows the results of frequency tests, i.e., isothermal segments of G′ and G’’, in the temperature range from −20 °C to 60 °C for (UNT) PIB 1 (Figure 3a,b) and PIB 2 (Figure 3c,d) samples, respectively. The G′ increased with increasing frequency and decreased with increasing temperature. This typical shape of curves shows that, in the low-frequency range, the molecular chains have enough time to react and start to disentangle. The result of this process is a reduction in storage modulus as time increases (decreased frequency). In the case of increased temperature, the thermal fluctuations, and the free volume increases, result in higher mobility of the molecules (faster relaxation process) [27] and consequently lower elastic modulus. At high frequencies, the molecular chains have no time to disentangle, and the storage modulus increases as the molecules respond as one entangled system. On the other hand, lowering the temperature reduces thermal fluctuations and free volume, which reduces the mobility and disables the process of chain disentanglement, resulting in the same effect on storage modulus as observed at high frequencies. To summarize, the increasing frequency or decreasing temperature have a similar effect on the polymer chain relaxation as they lead to lower mobility of the polymer chains. On the other hand, decreasing frequency or increasing temperature activate polymer chains relaxation, which increases molecular mobility. In the case of PIB as a main sealant in multi-layer insulating structures, temperature fluctuations can lead to functional failure. As temperature decreases, PIB material transitions from liquid-like to solid-like behavior, resulting in reduced sealing functionality. On the other hand, as temperature increases, the material transitions from solid-like to liquid-like behavior, which again leads to structural failure as the material could rupture already under small external loads. The viscous response of the samples was observed from the frequency-dependent loss modulus (Figure 3b,d) G″, which represents the factor proportional to dissipated mechanical energy during one loading cycle. G″ increased with increasing frequency and decreased with increasing temperature for both samples. The values regarding loss moduli were slightly higher for the PIB 2 sample at all temperatures, indicating a slightly higher consistency of this material compared to PIB 1, which was also observed during temperature sweep tests (Figure 2).

**Figure 4:**
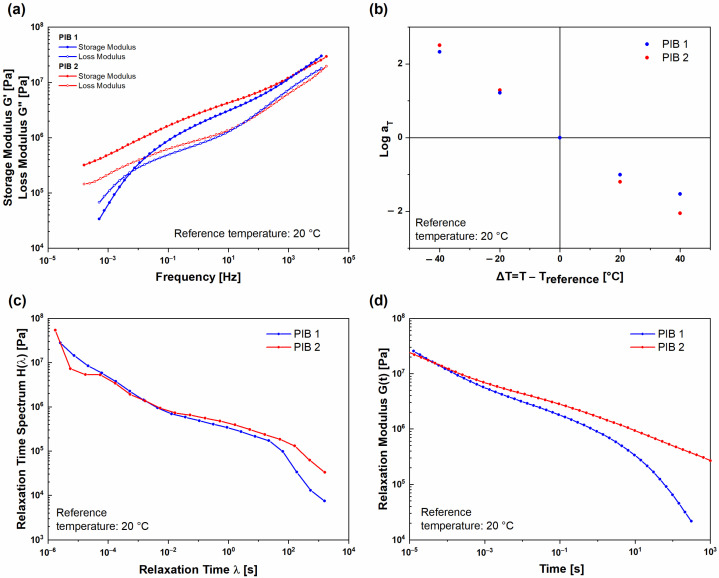
Comparison of (UNT) PIB 1 and PIB 2 samples at reference temperature of 20 °C: (**a**) viscoelastic properties, (**b**) horizontal shift factors, (**c**) relaxation time spectrum, and (**d**) time-dependent relaxation modulus.

To evaluate the behavior of the examined PIB materials through a wider frequency range and longer period of time, the frequency–temperature superposition was used to construct master curves at the reference temperature of 20 °C from isothermal segments dynamic moduli (Figure 4). For (UNT) PIB 1 and (UNT) PIB 2 samples (before the polymers were exposed to temperatures higher than 60 °C) the obtained master curves are presented in Figure 4a. A significant difference in frequency dependent viscoelastic behavior of the samples can be clearly observed, especially in the low frequency range. As previously observed, the PIB 1 sample exhibited lower values of dynamic moduli compared to PIB 2 sample in the whole frequency range examined. The difference was more pronounced in the frequency range below 1 Hz, where (UNT) PIB 1 entered the terminal region, exhibiting flow behavior. At longer times, the (UNT) PIB 1 sample exhibited unstable liquid-like behavior, which was, however, not observed for the (UNT) PIB 2 sample since the PIB 1 sample contains smaller molecular chains, which will be presented in the continuation. Furthermore, Figure 4b shows a temperature dependence of horizontal shift factors log a_T_ obtained during the construction of (UNT) PIB 1 and 2 master curves at a reference temperature of 20 °C. This so-called thermal shift function may be understood as the amount of temperature necessary to accelerate/decelerate the relaxation process. The results obtained indicate that high temperatures have a higher effect on the relaxation process of PIB 1 compared to PIB 2, which is in line with the generated master curves. For this reason, the relaxation time spectrum H(λ) was generated (Figure 4c), which, in addition to modelling the material behavior, describes the relaxation of molecular chains or groups of chains. The relaxation time λ may be understood as the response time of a certain molecular group (corresponding to their length), while the magnitude H may be understood as the contribution of this molecular group to the viscoelastic response (corresponding to their number). Considering this, it is clear that (UNT) PIB 2 has a higher number of long molecules than (UNT) PIB 1 as its H(λ) values are higher at longer relaxation times (Figure 4c). Moreover, the material with higher molecular weight would also exhibit a longer relaxation process since long molecules need more time to reconfigure, which may be observed through relaxation modulus G(t) (Figure 4d).

**Figure 5:**
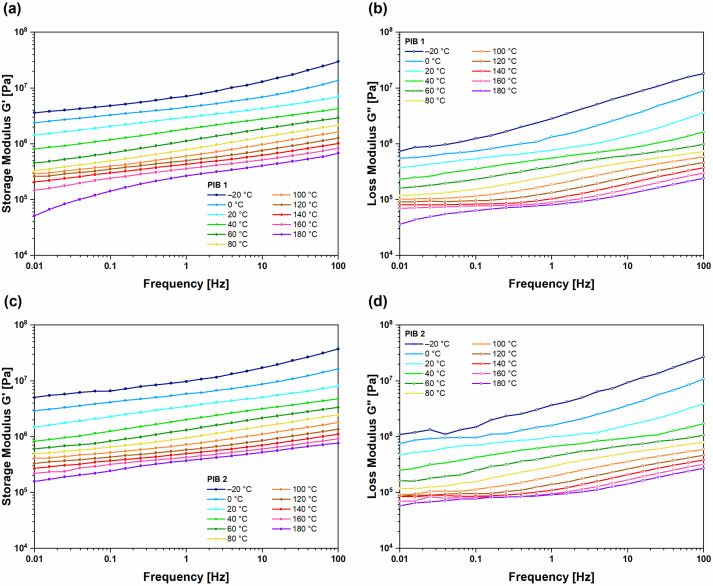
Isothermal segments of frequency-dependent dynamic moduli for (T) PIB 1 sample ((**a**) storage and (**b**) loss modulus) and (T) PIB 2 sample ((**c**) storage and (**d**) loss modulus).

As can be seen in the insert of Figure 2, the peaks of the tan δ curve were detected at about 80 °C, which indicates the temperature at which structural changes of PIB polymers started to occur. The changes in molecular structure affected the viscoelastic response of both PIB samples. In real application, the PIB as a primary sealant in multi-layer insulating glazing units is exposed to temperatures up to 110 °C. These temperatures can lead to different deformations of PIB sealants and therefore to disfunction of insulation products. To evaluate if we can avoid these deformations and the deterioration of the insulation during its lifetime, both samples were tempered for 10 min at the temperature of 180 °C to accelerate the structural changes. Figure 5 shows the frequency test results for tempered (T) PIB 1 and PIB 2 samples, respectively, in the temperature range from -20 to 180 °C. Similar to (UNT) samples, the storage modulus increased with increasing frequency and decreased with increasing temperature for both (T) samples. The (T) PIB 1 sample exhibited higher temperature dependence of storage modulus than (T) PIB 2. Moreover, the values of both moduli, i.e., storage and loss, were higher for PIB 2 compared to PIB 1. After the structural changes, the moduli of tempered (T) PIB 1 and (T) PIB 2 (Figure 5) were higher at the same temperature than before structural changes (Figure 3). Minor differences after structural changes were observed in the values of the loss modulus.

**Figure 6:**
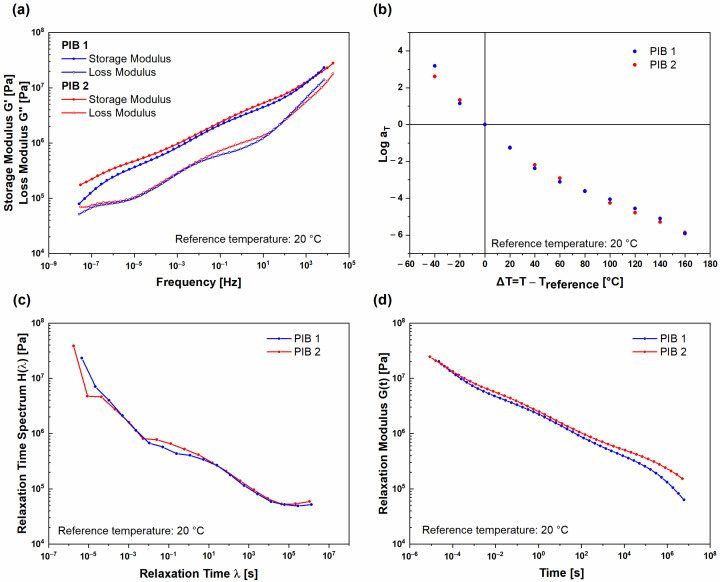
Comparison of (T) PIB 1 and PIB 2 samples at reference temperature of 20 °C: (**a**) viscoelastic properties, (**b**) horizontal shift factors, (**c**) relaxation time spectrum, and (**d**) time-dependent relaxation modulus.

By applying the frequency–temperature superposition principle, master curves for both (T) PIB 1 and 2 samples (after structural changes) were constructed at reference temperature 20 °C. The master curves are presented in Figure 6a. The results showed that, after structural changes, both the (T) PIB 1 and 2 samples exhibited very similar viscoelastic behavior throughout the examined frequency range. In the low frequency range, the storage modulus of (T) PIB 1 was slightly lower and decreased more significantly with decreasing frequency. Figure 6b presents the temperature-dependent horizontal shift factors log a_T_ determined during the construction of master curves. The results indicate that lower temperatures have a strong effect on the relaxation process for both PIB samples. The difference between log a_T_ of (T) PIB 1 and 2 samples is negligible. Moreover, the obtained results indicate that, after temperature conditioning of the PIB 1 and 2 samples, the structure changes resulted in a stable structure with rather comparable values of average molecular weight for both PIB materials. Furthermore, the relaxation time spectra H(λ) (Figure 6c) for both (T) PIB samples were very similar. Additionally, (T) PIB 2 exhibited a longer relaxation process (Figure 6d), which was attributed to the higher amount of molecular groups with relaxation times from 0.01 s to 10 s (Figure 6c).

**Figure 7:**
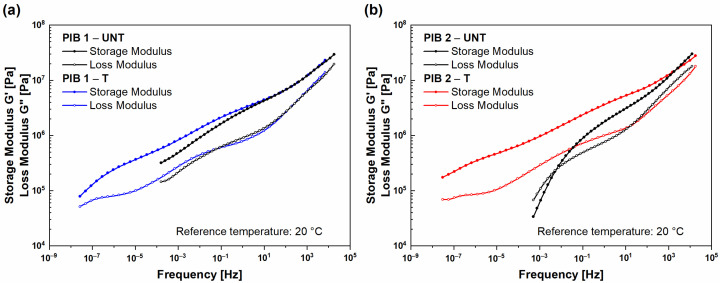
The comparison of viscoelastic properties (i.e., storage and loss modulus) before (marked -UNT) and after (marked -T) structural changing for (**a**) PIB 1 and (**b**) PIB 2.

The comparison of master curves for (UNT) and (T) samples PIB 1 and PIB 2 is presented in Figure 7. It can be seen that the differences in viscoelastic behavior of (UNT) and (T) samples for both PIB 1 and 2 materials are significant through a wide frequency range. After thermal treatment, the (T) PIB 1 and PIB 2 samples exhibited a solid-like response even at lower frequencies (longer times), which was observed as the flow behavior being shifted for over ~6 decades to lower frequencies (prevalence of G″ or decreasing of G′). From the molecular dynamics perspective, such a shift implies large structural changes, i.e., molecular growth, as larger molecules require longer times (excitation) at low frequencies to disentangle.

**Figure 8:**
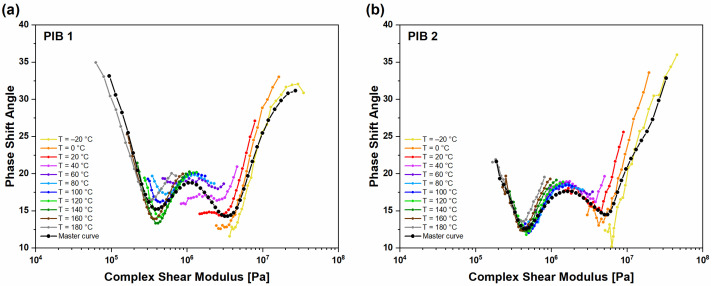
vGP plot of isothermal segments and constructed master curve at reference temperature for (**a**) PIB 1 and (**b**) PIB 2.

To evaluate the validity of the frequency–temperature superposition principle, Van Gurp–Palmen (vGP) plots were constructed from isothermal segments of phase shift angle δ vs. complex shear modulus G* (Figure 8). For thermo-rheologically simple materials (vGP), the plots should be temperature-independent [28]. The results confirmed that the vGP plots were temperature-independent, implying the thermo-rheological simplicity of PIB materials and justifying the use of applied analytical tools, i.e., superposition principles, spectral analysis, and obtained predictions.

**Figure 9:**
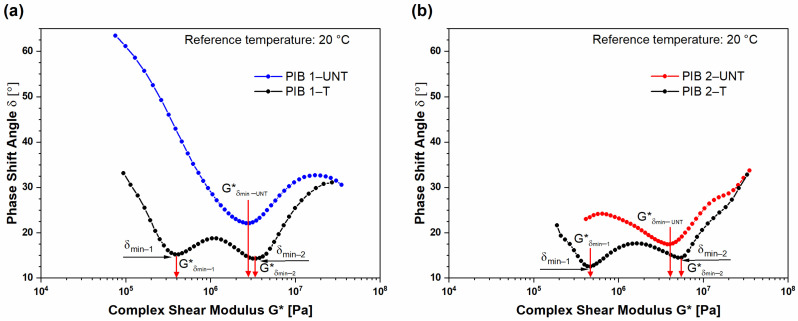
Van Gurp–Palmen plot. The dependence of phase angles of complex shear modulus before (UNT) and after (T) structural changes for (**a**) PIB 1 and (**b**) PIB 2. The reference temperature was 20 °C.

Furthermore, the vGP plots indicate the changes in molecular structure through the number and the position of local minimums. The values in phase shift angle at the minimum indicate the arm length and the width of molecular weight distribution, while the value of complex modulus $G_{N}^{0}$ at phase shift angle minimum (δ_min_) indicates the degree of branching, where higher $G_{N}^{0}$ values indicate higher branching of the molecules [24]. The values of complex modulus are usually determined at the intersection of two tangent lines from local minimum. Figure 9 presents the comparison of vGP plots for PIB 1 (Figure 9a) and PIB 2 (Figure 9b) before (UNT) and after (T) structural change, respectively. The results show that the phase shift angle (δ) was reduced after structural changes throughout the examined range of complex shear modulus. Moreover, the phase shift angle values of (T) PIB samples were lower compared to the values of (UNT) PIB samples, i.e., below 45°, indicating the domination of elastic behavior and higher degree of crosslinking [23,25]. Furthermore, the (UNT) PIB 1 and 2 samples exhibited a single minimum, indicating that the material is mostly composed of entangled linear polymer chains. It has already been reported that as many as 430 entanglements per chain can occur in PIB [26]. However, the lower value of complex modulus at local minimum for (UNT) PIB 1 (2.8 MPa) indicates lower molecular weight compared to (UNT) PIB 2, where the local minimum was determined at a complex modulus of 4.2 MPa. If we compare the two samples, we can see that the minimum value of δ for the (UNT) PIB 2 sample (δ_min_ = 17.3°) was lower compared to (UNT) PIB 1 (δ_min_ = 22.1°), which indicates a larger arm length and therefore wider molecular weight distribution in the PIB 2 sample.

**Figure 10:**
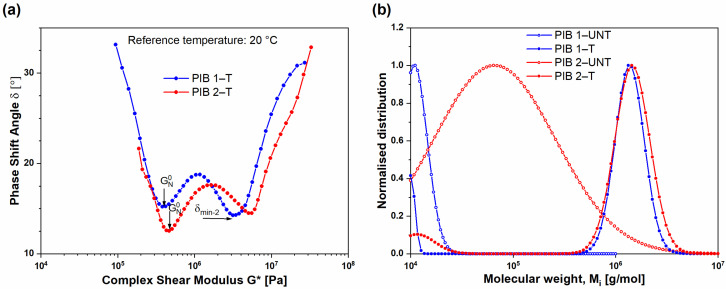
(**a**) Van Gurp–Palmen plots of tempered (T) samples; (**b**) molecular weight distribution for PIB 1 and PIB 2 samples before (UNT) and after (T) structural changes.

The results obtained show that, as the PIB materials were exposed to high temperatures, their structure changed, which was indicated by two local minimums as well as the transition from monomodal to bimodal molecular weight distribution (Figure 10). The comparison of vGP plots for both samples after structural changes is presented in Figure 10a. The first minimum for the (T) PIB samples was observed at complex modulus 0.4 MPa for PIB 1 and 0.45 MPa for PIB 2, which is in line with the wider molecular weight distribution of residual molecules for sample (T) PIB 2. Furthermore, the values regarding phase shift angle were smaller for (T) PIB 2 (δ = 12.5°) compared to PIB 1 (δ =15.2°), which indicates that the side chains of PIB 2 are larger. The second local minimum of tan δ could also be related to the degree of chemical reaction of various additives with PIB in the primary sealant. For the (T) samples, the second local minimum was observed at the values of complex modulus G*_δmin-2_ 3.6 MPa for PIB 1 and 5.4 MPa for PIB 2, respectively, which means that the (T) PIB 2 contains larger branched molecules. The values in phase shift angle in the second local minimum were very similar, i.e., 14.3° for (T) PIB 1 and 14.5° for (T) PIB 2; however, in the case of (T) PIB 1, the second δ_min_ was observed at lower values of complex shear modulus.

For detailed understanding and connection of previous results, the molecular weight distribution (MWD) was calculated from the relaxation time spectrum [29,30], generated from the frequency sweep tests from −20 °C to 180 °C. The molecular weight distributions before (UNT) and after (T) structural changes are presented in Figure 10b. A single peak of the molecular weight distribution was for (UNT) PIB 1 sample centered at 4.5 × 10^4^ g/mol, while, after structural changes ((T) PIB 1), an additional peak occurred at approximately two decades higher values, i.e., at 1.1 × 10^6^ g/mol. The same trend was observed for the PIB 2 sample: (UNT) PIB 2 sample exhibited a peak of molecular weight at 3.4·10^5^ g/mol, while the second peak for (T) PIB 2 was determined at 1.9 × 10^6^ g/mol. A narrower molecular weight distribution was observed for sample (UNT) PIB 1, where the full width at half maximum (FWHM) value was determined at 9.1 × 10^3^ g/mol. After structural changes, the (T) PIB 1 sample exhibited a wider molecular weight distribution with significantly higher FWHM value (1.1 × 10^6^ g/mol). The difference in the values of FWHM for PIB 2 sample was smaller; i.e., the FWHM for (UNT) PIB 2 was 3.2 × 10^5^ g/mol and for the (T) PIB 2 1.4 × 10^6^ g/mol, respectively. In the (T) PIB 1 and 2 samples, the bimodal molecular weight distribution was observed, where the peak at lower molecular weights represents a group of residual molecules, while the peak at higher molecular weights represents highly entangled branched molecules. One of the calculated parameters was also a polydispersity index (PDI), defined as a quotient of mass average molecular weight (Mw) to number-average weight (Mn). Before structural changes, the (UNT) PDI values of PIB 1 and 2 were determined to be 2.98 and 5.96, respectively. After structural changes (T), the PDI values of PIB 1 and 2 increased to 10.48 and 8.04, respectively. As observed from the PDI values, after structural change, the width of MWD increases as a result of branching. Moreover, due to increased temperature, the viscosity decreases, and the macromolecules can diffuse more easily through the phase, which enables the boosting of the intra-entanglement and consequently leads to higher molecular weight [31,32] of the (T) samples.

## 4. Conclusions

Within this paper, the thermal stability of two commercially available PIB materials, which are commonly used as primary sealants for energy-efficient multi-layer, i.e., six-pane, glazing units, was observed through polymer relaxation dynamics, i.e., viscoelastic properties. Understanding such behavior is imperative for achieving effective insulation in those elements for long-term use.

The results of temperature sweep tests showed that structural changes in PIB materials occur at ~80 °C. Below 80 °C, no structural changes were observed, and the materials remained thermo-rheologically simple, which is necessary for predicting their long-term viscoelastic response by the use of the time(frequency)–temperature superposition principle. However, as the temperature exceeded 80 °C, structural changes within materials occurred, and the materials lost their thermo-rheological simplicity. Nevertheless, the results show that the materials regained their simplicity when heated to 180 °C for 10 min.

In order to evaluate the long-term viscoelastic response as well as thermally induced structural changes within PIB materials, frequency sweep tests were conducted on untempered (UNT) and tempered (T) samples. From the obtained isothermal segments of viscoelastic properties (G′ and *G*″), master curves were constructed at a reference temperature of 20 °C using the frequency–temperature superposition principle. From the obtained master curves, relaxation time spectrum, vPG plots, and the molecular weight distribution of examined materials were determined. The master curves of G′ and G″ as well as the relaxation time spectrum of PIB materials indicate that the UNT samples exhibit a disentangled structure and viscous-like behavior, while the (T) samples, exhibit a more branched comb-like structure with solid-like behavior, extending the material response for ~6 decades towards lower frequencies, i.e., longer time periods. Further structural changes were identified by observing vPG plots and molecular weight distributions, indicating that, during thermal treatment, the structure of PIB materials changes from linear entangled to branched comb structure. Moreover, molecular weight distributions transition from monomodal to bimodal (groups of residual and branched molecules) and shift toward higher molecular weights. It was undoubtedly shown that, in order to use the PIB sealants in multi-layer six-pane glazing units, where the temperatures can exceed 100 °C, the PIB should be thermally conditioned prior to its utilization. Only in this way is the structure of the material stabilized, which significantly improves the long-term behavior of this material.

The presented results are of key importance not only for optimizing the processing and working conditions of sealants during their application and utilization but can also be directly used to simulate the mechanical responses of the sealants using currently available FEM software packages to predict their functional and structural lifetime.

## Data Availability

Data are contained within the article.
